# Longitudinal Change in Sleep and Daytime Sleepiness in Postpartum Women

**DOI:** 10.1371/journal.pone.0103513

**Published:** 2014-07-31

**Authors:** Ashleigh J. Filtness, Janelle MacKenzie, Kerry Armstrong

**Affiliations:** Centre for Accident Research and Road Safety – Queensland, Queensland University of Technology, Kelvin Grove, Queensland, Australia; University of Alabama at Birmingham, United States of America

## Abstract

Sleep disruption strongly influences daytime functioning; resultant sleepiness is recognised as a contributing risk-factor for individuals performing critical and dangerous tasks. While the relationship between sleep and sleepiness has been heavily investigated in the vulnerable sub-populations of shift workers and patients with sleep disorders, postpartum women have been comparatively overlooked. Thirty-three healthy, postpartum women recorded every episode of sleep and wake each day during postpartum weeks 6, 12 and 18. Although repeated measures analysis revealed there was no significant difference in the amount of nocturnal sleep and frequency of night-time wakings, there was a significant reduction in sleep disruption, due to fewer minutes of wake after sleep onset. Subjective sleepiness was measured each day using the Karolinska Sleepiness Scale; at the two earlier time points this was significantly correlated with sleep quality but not to sleep quantity. Epworth Sleepiness Scores significantly reduced over time; however, during week 18 over 50% of participants were still experiencing excessive daytime sleepiness (Epworth Sleepiness Score ≥12). Results have implications for health care providers and policy makers. Health care providers designing interventions to address sleepiness in new mothers should take into account the dynamic changes to sleep and sleepiness during this initial postpartum period. Policy makers developing regulations for parental leave entitlements should take into consideration the high prevalence of excessive daytime sleepiness experienced by new mothers, ensuring enough opportunity for daytime sleepiness to diminish to a manageable level prior to reengagement in the workforce.

## Introduction

With the birth of every infant the new mother must adjust to the demands of parenting; one component is a requirement to remain “functional” while experiencing potentially severe sleep disruption. Sleep disruption strongly influences daytime functioning including the capacity to deal with the unexpected, changing situations and distractions, as well as the ability to evaluate risks [1]. There is a long history of investigation into the implications of sleep loss and sleepiness of vulnerable populations including shift-workers and commercial drivers [2], [3], however, in comparison postpartum women are largely overlooked.

Childbirth is an extraordinary, everyday experience; in the year 2011, 301 617 infants were born in Australia [4] and 5 229 813 infants were born in the EU [5], resulting in countless potential occurrences of postpartum sleep loss and subsequent daytime sleepiness. Standard sleep research techniques, including sleep diaries and subjective measures of sleepiness have successfully been used to investigate sleepiness in postpartum mothers [6]–[11]. In this series of investigations Insana, Montgomery-Downs and co-authors reported on the interaction between daytime sleepiness, nocturnal sleep and neurobehavioral performance using a combination of subjective and objective measures. In brief, their correlational findings report that daytime sleepiness (measured by the Epworth Sleepiness Scale [ESS]) was lower during postpartum week 13 than in week 7, and lower in postpartum week 7 than week 2 [8]. Despite reduction in ESS over the initial weeks, postpartum women were sleepier compared with control women [7]. In particular, reaction time to the Psychomotor Vigilance Test (PVT) was slower in primiparous women compared with nulliparous control women throughout the first 12 postpartum weeks [7]. Additionally, postpartum women had a significantly shorter sleep onset latency (measured by Multiple Sleep Latency Test [MSLT]) than control women [9]. Interestingly, sleep disruption rather than total sleep obtained was influential in daytime sleepiness [6]. Additionally, subjective sleepiness (measured by Stanford Sleepiness Scale [SSS]) was most associated with sleep quality [8]. The quantity of sleep obtained by new mothers from postpartum weeks 2 to 16 was relatively consistent (7.2 hours). However, sleep quality improved over the same time period due to a reduction in sleep fragmentation and increase in sleep efficiency [10]. Furthermore, while both subjectively and objectively (Actigraphy) recorded sleep times were associated with daytime sleepiness this relationship differed and changed over time [8], demonstrating a unique importance of *subjective* as well as *objective* sleep measures.

Throughout the night maternal sleep is regularly interrupted in response to the needs of their infant. Compared to the last month of pregnancy, during the first postpartum month mothers have less night time sleep and spend a greater time awake following sleep onset [12]. This is to be expected as initially infants lack a regular circadian rhythm, which must develop over time, and in many infants is established by postpartum week 12 [13]. At one month postpartum infants may be expected to sleep for an average of 3 to 4.5 hours in a single episode [14]. Despite the fragmentation to maternal sleep there is research evidence to suggest that the total amount of sleep obtained is similar to the general population [10], [15] as new mothers extend their night time sleep period and/or nap during the day. Understanding and mitigating the impact of this sleep disruption is important for the health of the mother, as a large amount of wake after sleep onset and low sleep efficiency are predictive of postpartum fatigue severity and mood in general [16], [17]. The level of sleep disruption experienced is reportedly surprising to new monthers [18], suggesting they may be ill-prepared for its consequences.

Although important evidence regarding the characteristics and implications of sleep disturbance during the postpartum period is starting to emerge, there is still a lack of information regarding longitudinal change during this period and how this impacts daytime sleepiness. In particular, the current study addresses this gap in the scientific knowledge by following the same healthy women over time in a repeated measures design. Previous studies have tended to compare sleep and sleepiness at different postpartum weeks between different groups of mothers or to focus exclusively on those mothers with postpartum depression and/or postpartum fatigue. In addition, the current work considers the postpartum period up to week 18, which is longer than typical in studies of new mothers. This study aimed to quantify longitudinal changes in sleep duration, night time disturbance and daytime sleepiness of a sample of Australian mothers during postpartum weeks 6, 12, and 18. It was hypothesised that; (1) total sleep time would be consistent across time points; (2) sleep disturbance would decrease across time points; (3) daytime sleepiness would be prevalent but decrease across time points; and (4) daytime sleepiness would be correlated with both sleep quantity and sleep quality.

## Methodology

### Procedure

To examine change in sleep disturbance and daytime sleepiness in postpartum women, a longitudinal study protocol was undertaken. Participants maintained self-administered sleep-wake diaries (see File S1) completed each day for seven consecutive days at three time points: 6, 12, and 18 weeks following the birth of their child. A phone call was made prior to mail-out to let the participant know the diary was arriving and to encourage participants to contact the research team if they had any questions. A follow-up phone call was made once the diary was returned to the researchers to say thank you and to see if there were any difficulties in filling out the materials. All participants received clear instructions to record their sleep each day after waking, rather than completing all entries at the end of the week.

### Measures

#### Sleep

At each time point participants completed a self-administered paper based diary. Each diary represented 168 hours (7 days) in 7 separate bars, divided into hours of the day. Participants were asked to mark all sleep episodes on the bar, including naps, for themselves and their infant. These were coded in 15 minute epochs. Additionally, the location of sleeping for both mother and infant was recorded as well as any reasons for waking during the night time sleep period.

#### Sleepiness

All participants received instructions on how to distinguish between sleepiness and fatigue. Sleepiness was defined as resulting from not having had enough sleep, while fatigue was defined as physical or emotional exhaustion. Subjective sleepiness was assessed using the Karolinska Sleepiness Scale (KSS) [19]. This nine point scale ranges from 1 = very alert to 9 = very sleepy, it has been validated and reflects physiological signs of sleepiness in healthy populations [19], [20]. Each day participants reported how sleepy they felt when they woke up using the KSS. Scores of 6 or more are indicative of feeling sleepy. Sleepiness was also examined using the Epworth Sleepiness Scale (ESS) [21], completed once at each time point, on the final day of the assessment period (sleep diary day 7). Participants rate their susceptibility to falling asleep using a scale from 0 to 3, in relation to eight real life scenarios. Scores are totalled with those ≥12 indicative of excessive daytime sleepiness (EDS). The ESS was initially developed and validated as a measure of trait sleepiness; however, it has recently been successfully used to reflect sleepiness change over time with healthy women during the postpartum period [8].

### Statistical analyses

Seven consecutive days of diary entries were required at each time point. This includes five *working* days and two *weekend* days. The full seven day report was required from each participant on each occasion. The nocturnal total sleep time (TST) was calculated as the total number of minutes sleep obtained during the night. This contained all sleep episodes when any intermittent wake periods were shorter than the subsequent sleep episode. By identifying nocturnal sleep in this manner all nocturnal sleep was considered regardless of the exact hours of the day it occurred between. Daytime sleep represents napping behaviour. Data entries from each day were used to calculate each participant’s weekly mean for sleep quantity and sleep quality. Sleep quantity measures were: TST and daytime sleep duration. Sleep quality measures were: wake duration after sleep onset (WASO), number of nocturnal wake episodes, duration of the first nocturnal sleep period, longest nocturnal sleep period and a sleep disturbance index (SDI; SDI = (WASO/TST)*100). This SDI has previously been used to quantify sleep disturbance of obstructive sleep apnoea patients when continuous positive airway pressure (CPAP) treatment is withdrawn [22] and to confirm equivocal sleep disturbance for CPAP treated obstructive sleep apnoea patients compared with healthy controls [23].

Data were analysed using SPSS 20.0 statistical software. An alpha level of.05 was used to determine statistical significance. Dependent variables were analysed using a repeated measures ANOVA with the within-subjects factors of *time* (3 levels: week 6, week 12, and week 18). Huynh–Feldt adjustments were used if the assumption of sphericity was not met. Post-hoc pairwise comparisons were conducted using Bonferroni tests. To supplement the interpretation of the results, partial η2 was used as an estimate of effect size. Bivariate correlations were calculated between four overnight sleep measures and daytime sleepiness measures at each time period, p value significance was adjusted accordingly (p<.0125).

Means and standard errors are reported unless otherwise stated. The data set is provided as File S2.

## Results

### Participants

Forty-four new mothers, mean age 30.1 years (SD = 4.0), who had healthy infants were recruited for the study through advertisements at the university and in a local newspaper. Participants were recruited both before and after the birth of their child; 16 of the participants who completed the study were recruited prior to the birth of their child. Participants recruited post birth signed up for the study during postpartum weeks 1 to 5. All prospective participants underwent screening using an in-house telephone questionnaire to ensure they were free from psychiatric (including no recent history of depression) or sleep related medical problems, and had a healthy infant. Additionally, mothers who had undergone birth by caesarean section or had a multiple birth were excluded. In total, 8 prospective participants were excluded during screening due to caesarean section or premature birth. Due to the longitudinal nature of the study and the commitment required, 11 participants withdrew from the study. No significant differences were identified between those who withdrew and those who completed the study.

Analyses presented in this paper are based on data from the fully completed sleep diaries of 33 postpartum women (16 primiparous), mean age 30.0 years (SD = 4.0). This is reflective of the recent mean age of Australian mothers giving birth (30.6 years) [4]. Independent t tests confirmed no significant differences for nocturnal sleep time or subjective sleepiness between mothers with one or multiple children. Consequently, data from primiparous and multiparous participants are considered together. All participants were married or in a de-facto relationship at the start of the protocol and were non-smokers. The majority of participants (n = 26) were highly educated, having a Bachelor’s degree or higher qualification. All participants provided written informed consent and the study was approved by the Queensland University of Technology Human Ethics Committee.

### Infant care behaviours

The majority of participants were exclusively breastfeeding [Week 6: 81.8%, Week 12: 72.7%, Week 18: 60.6%]. No effect of breastfeeding on any sleep or sleepiness measures was apparent. The proportion of participants exclusively providing all night time infant care remained consistent [Week 6: 27.3%, Week 12: 24.2%, Week 18: 30.3%] and had no apparent effect on any measures of sleep or sleepiness.

### Nocturnal sleep

**Figure 1:**
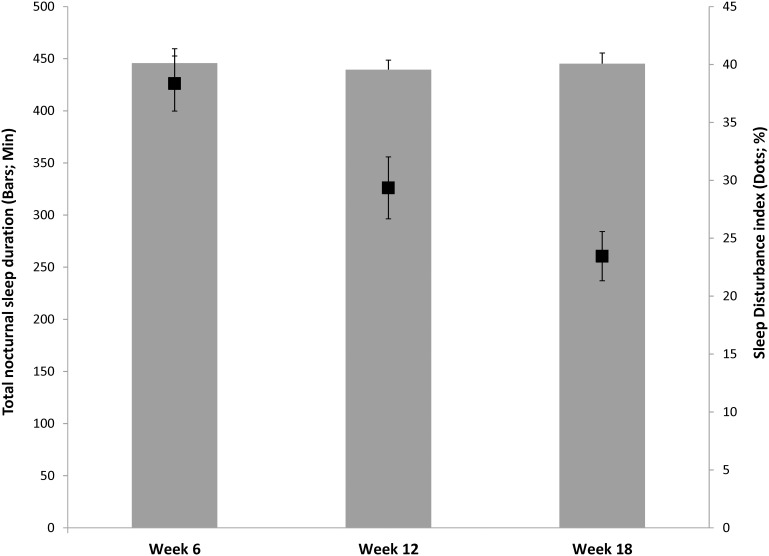
Postpartum nocturnal sleep duration (minutes) and sleep disturbance index (%), error bars represent standard error.

Changes to sleep quality and quantity are displayed in Table 1. Across the study period there was no significant change in the TST [*F*(1.58, 50.58) = .227, *p* = .745, partial η2 = .007] or the duration of the initial nocturnal sleep episode [*F*(1.77, 56.47) = 1.923, *p* = .160, partial η2 = .057]. Further, the frequency of wakings during the nocturnal sleep period also remained consistent [*F*(1.70, 54.46) = 1.907, *p* = .164, partial η2 = .056]. The consistency in TST appeared to be facilitated by either earlier bed times or later final wake up times during the earlier assessment time points. Despite the consistency of TST and frequency of wakings, the duration of the longest continuous nocturnal sleep episode significantly increased over *time* [*F*(1.75, 55.86) = 4.364, *p* = .021, partial η2 = .120]. Accordingly, the duration of WASO decreased in a linear fashion across the study points, with post hoc analysis revealing each time point to be significantly different than the other two. Consequently, there was a similar significant reduction of SDI [*F*(1.77, 56.56) = 15.204, *p*<.001, partial η2 = .322]. TST and SDI are displayed in Figure 1.

**Table 1:** Change in postpartum maternal sleep and sleep disturbance.

	Week 6	Week 12	Week 18	*F*
**Nocturnal total sleep time (minutes)**	445.84 (13.71)	439.55 (9.01)	445.32 (10.15)	0.227
**Initial nocturnal sleep episode (minutes)**	231.57 (14.07)	256.92 (14.73)	265.49 (18.32)	1.923
**Longest nocturnal sleep episode (minutes)**	246.30 (12.83)	273.70 (13.31)	292.66 (16.00)	**4.364** *
**Wake after sleep onset (minutes)**	106.75 (7.81)	77.86 (7.94)	59.67 (5.17)	**14.059** **
**Sleep disturbance index (%)**	38.36 (2.37)	29.35 (2.68)	23.45 (2.13)	**15.204** **
**Wakings during nocturnal sleep period (n)**	1.90 (0.90)	1.65 (0.14)	1.70 (0.16)	1.907
**Day time sleep (minutes)**	50.00 (14.85)	28.70(13.35)	14.92 (8.26)	2.301

**p<.001,

*p<0.05.

### Daytime sleep

Across the study period there was a trend for a reduction in daytime sleep [*F*(1.19, 38.05) = 2.301, *p* = .134, partial η2 = .067]. Napping behaviour appeared to be driven by individual preference with some participants napping frequently and others not at all. Consequently, large standard deviations render this trend not significant.

### Daytime sleepiness

**Figure 2:**
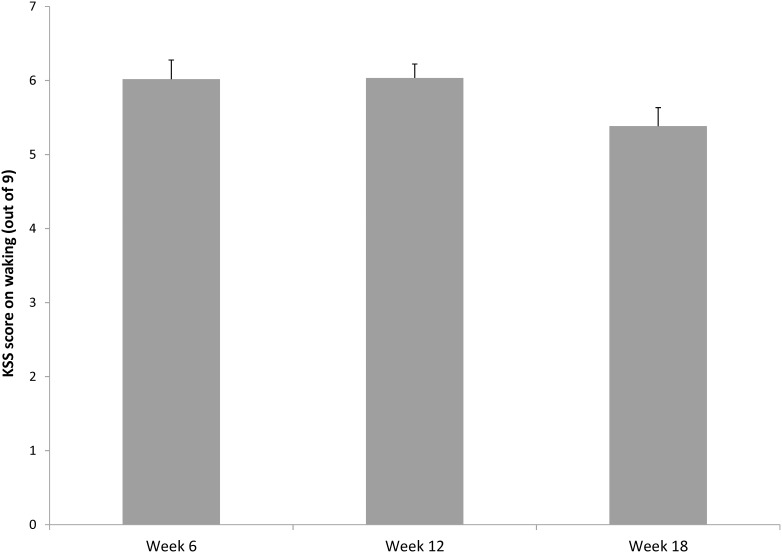
Postpartum daytime sleepiness, KSS score on waking, error bars represent standard error.

Three participants failed to complete the KSS consistently at each time point; consequently KSS results are presented for 30 participants only. Figure 2 presents the effect of *time* on KSS. There was a significant main effect of *time* on KSS [*F*(2, 58) = 5.090, *p* = .009, partial η2 = .149]. Pairwise comparison identified waking sleepiness experienced at 18 weeks [mean = 5.36, SE = 0.27] to be significantly lower than waking sleepiness experienced at 12 weeks [mean = 6.01, SE = 0.20, *p* = .019]. Accordingly, the proportion of participants reporting feeling sleepy when they woke (KSS ≥6) was similar for Week 6 (54.55%) and Week 12 (57.58%), and then reduced in Week 18 (30.30%).

**Figure 3:**
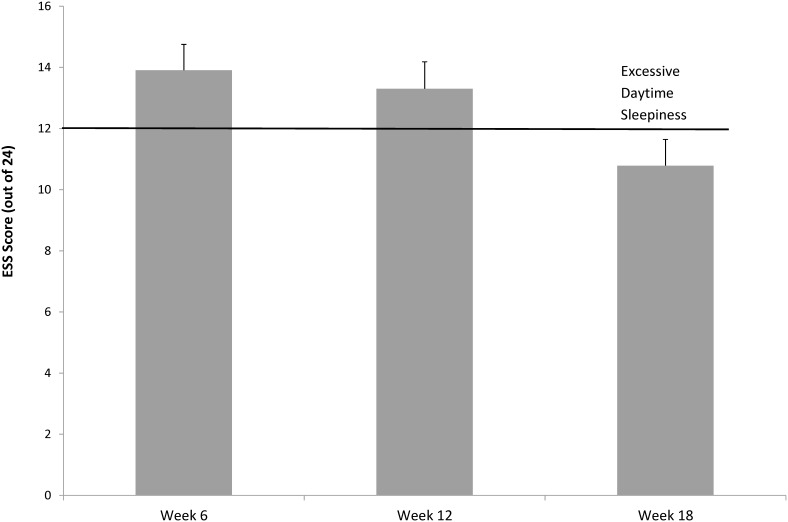
Postpartum Epworth Sleepiness Scale, error bars represent standard error.

Across the three time points there was a significant change in ESS [*F*(1.59, 50.97) = 15.697, *p*<.001, partial η2 = .329], presented in Figure 3. Pairwise comparisons identified ESS at 18 weeks [mean = 10.79, SE = 0.87] to be significantly lower than at either 6 weeks [mean = 13.91, SE = 0.84, *p*<.001] or 12 weeks [mean = 13.30, SE = 0.86, *p = *.002]. In accordance, the proportion of participants experiencing EDS (ESS ≥12) was the same at Week 6 and Week 12 (69.70%), which reduced at Week 18 (54.54%).

### Associations between nocturnal sleep and daytime sleepiness

Table 2 displays the outcome of correlation analysis for KSS and sleep measures. KSS was only significantly correlated to sleep quantity (TST) at week 18 [*r* ^2^ = −.528, *p* = .002]. In contrast, KSS was correlated with SDI at Week 6 [*r* ^2^ = .516, *p* = .003] and Week 12 [*r* ^2^ = .476, *p* = .006], although not at Week 18 [*r* ^2^ = .394, *p* = .023]. At the two earlier time points KSS was significantly correlated with both duration of the initial nocturnal sleep period [Week 6: *r* ^2^ = −.545, *p* = .002; Week 12: *r* ^2^ = −.475 *p* = .006] and duration of the longest nocturnal sleep period [Week 6: *r* ^2^ = −.506, *p* = .002; Week 12: *r* ^2^ = −.449, *p* = .010] but not TST [Week 6: *r* ^2^ = −.352, *p* = .056; Week 12: *r* ^2^ = −.207, *p* = .257]. ESS was not significantly correlated with any sleep measure.

**Table 2:** Correlation between KSS and nocturnal sleep metrics.

	Week 6	Week 12	Week 18
**Total sleep time**	−.352	−.207	−**.528** *
**Initial sleep period**	−**.545** *	−**.475** *	−.186
**Longest sleep period**	−**.506** *	−**.449** *	−.303
**SDI**	**.516** *	**.476** *	.394

*denotes significance adjusted p<0.0125.

## Discussion

The quantity of sleep obtained by healthy new mothers remains consistent across postpartum weeks 6, 12, and 18. With a TST of approximately 7 h 20 m, Australian new mothers obtained more sleep than the average American worker (6 h 53 m) [24]. This is very similar to an American study following new mothers from postpartum weeks 2 to 16, where average TST was 7.2 hours [10]. This provides a cross-cultural replication of the finding that postpartum women experience disturbed sleep, but not necessarily reduced total sleep time. Inevitably, new mothers will wake in the night to attend to their infant, and the number of times per night appears to remain consistent during the first 18 postpartum weeks. This study is one of the first to follow the same healthy new mothers through the first 18 postpartum weeks. Findings are in line with previous findings obtained from comparing different groups of new mothers, where each time point (weeks 2, 7 and 13) was analysed independently [8]. SDI significantly reduced over time, apparently driven by a reduction in WASO minutes, suggesting improved efficiency by mothers at settling their infant back to sleep and/or development of the infant’s circadian rhythm. Furthermore, subjective sleepiness (KSS) at the earliest two time points correlated to SDI but not TST. These two findings highlight the importance of sleep quality as opposed to sleep quantity, especially during the first 12 weeks. In particular, it appears that during the early postpartum period, when sleep disturbance is greatest, the duration of the initial sleep period and longest continuous sleep period are key factors in influencing daytime sleepiness. Later in the postpartum period, when WASO is reduced, TST becomes the sleep measure associated with daytime sleepiness.

Despite the substantial and stable TST, EDS was reported by the majority of participants, with over 50% of participants still reporting EDS at week 18. To contextualise this finding, consider a clinician’s response to an individual presenting with an 18 week history of EDS. Within Australia, the ESS is a recommended tool for assisting clinical decision regarding patient fitness to drive [25]. Using this tool the clinician would likely offer advice regarding implications for driving and daytime impairment. Interestingly, the current study found no correlation between ESS and nocturnal sleep. This is in contrast to a previous regression analysis including ESS during postpartum week 11, where ESS was found to be independently associated with TST [6]. However, participants in the current study reported greater ESS following similar sleep time (Week 12: ESS = 13.3, TST = 439.5 min) compared with the previous study (Week 11: ESS = 8.2, TST = 436.1 min). Little is known about the implications for EDS in healthy postpartum women, however, the clinically relevant amount of EDS within the current study population and reported slowed reaction times of postpartum women compared to controls [7] demonstrate that this is an important area requiring future research.

By week 18, only 5 participants were working full-time. This is likely influenced by Australian legislation which provides parental leave support for up to 18 weeks [26]. EU mothers have similar benefits, being entitled to four months maternity leave [27]. In contrast, American mothers receive 12 weeks unpaid leave [28]. Discrepancies between jurisdictions will likely impact the proportion of mothers returning to the workplace whilst experiencing clinically relevant daytime sleepiness. Despite recognition of the potential dangers resulting from sleepy employees [29] there has been little attempt to understand sleepiness in new parents returning to work. This oversight within the literature has further consequences when considered that despite high levels of sleepiness, new mothers “persevere” to meet their essential work and economical demands [30]. Future research should consider practical implications for high risk activities, such as driving, and the potential impact of self-limiting risk exposure behaviours.

Postpartum mothers are a unique group of otherwise healthy individuals from whom much can be learnt about the effects of sleep disturbance. However, investigations into sleep within the postpartum period have traditionally been restricted to the strong association between sleep and postpartum depression (PPD) [31]–[33]. In particular, sleep duration <6 h in a 24 h period appears associated with PPD [32], and wake after sleep onset and low sleep efficiency are predictive of postpartum fatigue severity [16]. The current findings, with healthy participants, emphasise the importance of sleep disturbance over sleep quantity. With reportedly 16.5% of new mothers experiencing PPD [33] future investigation into SDI within this group could provide valuable understanding.

All data in the current study were collected by self-report. This methodological approach is important because perception of sleep quality in new mothers appears to have a central role in the experience of daytime sleepiness. For example, self-reported sleep quality more consistently accounts for subjective daytime sleepiness than actigraphy recorded TST [8]. Furthermore, there is a stronger association between new mothers subjective sleep quality and mood disturbance than between actigraphy measured TST and mood [17]. However, because all data were collected using a field-based protocol, participants were unsupervised during instrument completion. All participants received clear instructions to complete the diary each day after waking; although, there was no objective facilitation of this. Nonetheless, the field-based design provides ecological validity to results. To minimise participant burden the number of diary entries was kept to as few as possible. Consequently, participants did not regularly record KSS throughout the day; therefore circadian changes cannot be evaluated. At week 18 over half of the participants were still experiencing EDS. Future research should consider an extended study protocol evaluating the prolonged experience of EDS. The small sample size, as well as ethnicity and education level homogeneity limits the generalisability of the research findings. Future research may wish to investigate this topic with a broad range of participants in order to investigate potential differences due to social demographic factors. In addition, future research may wish to consider the role of fathers in infant care and the impact on their sleep and sleepiness. In particular, new fathers who are participating in night time infant care and going to work during the day may be at increased risk of workplace and other accidents due to daytime sleepiness. The first data collection point was during postpartum week 6 because it was considered unethical to request new mothers to participate in this relatively time consuming protocol at any earlier time point. Consequently, the current study provides no information on the first 5 postpartum weeks. As the current work considers only subjective measures of sleep and sleepiness, findings cannot be inferred to objective measures. Future research may also wish to consider the longitudinal relationship between subjective and objective measures of sleep in postpartum mothers.

Using a longitudinal, field-based protocol this study is one of the first to follow new mothers during the initial 18 postpartum weeks. Daytime sleepiness is common and is influenced by night time sleep disturbance (WASO and SDI) rather than total quantity of sleep achieved. EDS can reach clinically significant levels posing implications for safety-critical situations, such as driving. The dynamic nature of sleep during this postpartum period should be taken into account when designing interventions to address sleepiness. For example, a structured remote-learning information program would be useful during the late stages of pregnancy for first time mothers. Such a program could include details about how sleep may be expected to change during the postpartum period and advise on putting coping strategies in place. This would be particularly beneficial as many new mothers are surprised by the level of sleep disruption experienced during this period [18]. Policy makers should consider EDS when determining the minimum parental leave entitlement, and aim to ensure that new parents take adequate time away from the workplace for daytime sleepiness to diminish to a non-critical level.

## Supporting Information

File S1 Self-administered sleep diary.
(DOC)

File S2 Data set.
(XLSX)
