# Supplementary material for: Longitudinal Change in Sleep and Daytime Sleepiness in Postpartum Women
Source: PLoS One. 2014 Jul 31;9(7):e103513. doi: 10.1371/journal.pone.0103513 (PMC4117520; doi:10.1371/journal.pone.0103513)
Supplement: Supplementary file 1 [file pone.0103513.s001.doc]

ID: ____________________

Week: __________________

Queensland University of Technology
Brisbane Australia

CARRS

Postpartum Fatigue Questionnaire

The following questionnaire will ask you some questions about you, your overall sleepiness and well being, as well as some of the incidents that may have occurred to you recently. Please answer all the questions as honestly as possible. The information you provide will be used to develop education material for new parents to help combat fatigue.

Please fill out this questionnaire *on the day* that you complete your weekly sleep diary.

Please note that when we use the word *sleepiness* in this questionnaire we are referring to feeling of not having had enough sleep. When we use the term *fatigue* we refer to the feeling of being physically or emotionally worn out or exhausted which may come from getting through your daily tasks, being busy, or performing new or challenging tasks.

Section 1: About You

Please answer the following questions by writing your answer in the blank space provided, or ticking the appropriate box.

Q1. What is your age in years? ____________years

Q2. What is your current marital status?

Married/De Facto/ Partner Single
Widowed Divorced or Separated

Q3. Do you have other children living in your home other than your new baby?
Yes No If yes, please list their ages. __________________

Q4a. What is your current employment status?

Full Time Part Time Casual Volunteer

Student Unemployed Primary Carer

Q4b. Are you currently on Maternity leave Yes No
If so, how long is your leave? __________________ months

Q5. What is your highest education level?

Junior Certificate Senior Certificate Associate Diploma

Diploma Degree Honours Degree Masters or PhD

Q6. Are you currently taking any prescription medications? Yes No
If yes, please list medication, number taken per day and dosage per tablet in mg.
____

Q7. Are currently taking any natural or over the counter medications?
Yes No
If yes, please list number taken per day and dosage per tablet in mg.
____

Q8. Do you smoke Yes No
If yes, how many cigarettes per day? ______

Q9. Do you consume alcohol? Yes No
If yes, please estimate how many standard drinks consumed in a week.
____

Section 2: About your baby and the support you have for your baby's care

Q1. Tick the box that best describes how you feed your child.

Fully breastfeed

Combination breast/bottle feed

Bottle feed

Solids and Breast

Solids and Bottle

Q2. Do you have any help with the night time care of your baby?

No one
Spouse/Partner
Other family member
Paid help
Other ____________

Q3. How often do you receive help with night time care of your baby?

Everyday 1-2 times a week 1-2 times a month

Less than once a month Never

Q4. Do you have any help with the day time care of your baby?

No one
Spouse/Partner
Other family member
Friends
Paid help
Formal Child Care
Other ____________

Q5. How often do you receive help with the day time care of your baby?

Everyday 1-2 times a week 1-2 times a month

Less than once a month Never

Section 3: About How You Are Feeling

Q1. How likely are you to doze off or fall asleep in the following situations, in contrast to feeling just tired? This refers to your usual way of life in recent times. Even if you have not done some of these things recently try to work out how they would have affected you.

Use the following scale to choose the most appropriate number for each situation:

0 = no chance of dozing

1 = slight chance of dozing

2 = moderate chance of dozing

3 = high chance of dozing

Situation	Chance Of Dozing			
	**no chance**	**slight chance**	**mod chance**	**high chance**
Sitting and reading	0	1	2	3
Watching TV	0	1	2	3
Sitting inactive in a public place (e.g. a theatre or a meeting)	0	1	2	3
As a passenger in a car for an hour without a break	0	1	2	3
Lying down to rest in the afternoon when circumstances permit	0	1	2	3
Sitting and talking to someone	0	1	2	3
Sitting quietly after a lunch without alcohol	0	1	2	3
In a car, while stopped for a few minutes in traffic	0	1	2	3

That's All

Thank you for taking the time to fill out the questionnaire

ID: ______________________________

SLEEP-WAKE DIARY

Instructions

INSTRUCTIONS:

1. Please fill out the sleep-wake dairy every day (first thing in the morning is best). Use a pen or pencil to ‘colour in’ the hours that you and others in your house had a sleep (even a short nap). Try to do this to the nearest half an hour.

For example -

Noon yesterday | Midnight | This morning

	8 am	9 am	10 am	11 am	12 ☼	1 pm	2 pm	3 pm	4 pm	5 pm	6 pm	7 pm	8 pm	9 pm	10 pm	11 pm	12 ❍	1 am	2 am	3 am	4 am	5 am	6 am	7 am	8 am	**Location?**
You																										**In my bed**
Baby																										**In her cot**

DAY 1

(Note: this page was replicated for each of the 7 days)

1. What is the day today?

2. Mark all sleep periods (including naps) for you and your baby, your partner and for any other children in the house:

Noon yesterday — Midnight — This morning

	8 am	9 am	10 am	11 am	12 ☼	1 pm	2 pm	3 pm	4 pm	5 pm	6 pm	7 pm	8 pm	9 pm	10 pm	11 pm	12 ❍	1 am	2 am	3 am	4 am	5 am	6 am	7 am	8 am	**Location?**
You																										
Baby																										

What woke you up?

3. How long did it take you to fall asleep (main sleep episode)?

4. How sleepy did you feel when you woke up this morning?

①	②	③	④	⑤	⑥	⑦	⑧	⑨
Very alert		Alert		Neither alert nor sleepy		Sleepy		Very sleepy
